# Negative correlation between endoglin levels and coronary atherosclerosis

**DOI:** 10.1186/s12944-021-01545-2

**Published:** 2021-10-03

**Authors:** Haibin Chen, Yiping Wang, Bing Sun, Xunxia Bao, Yu Tang, Feifei Huang, Sibo Zhu, Jiahong Xu

**Affiliations:** 1 Department of Cardiovascular, Tongji Hospital, School of Medicine, Tongji University, No. 389, Xincun Road, Putuo District, 200065 Shanghai, China; 2 School of Life Science, Anhui Medical University, Hefei, 230022 China; 3 Department of Epidemiology, School of Public Health, State Key Laboratory of Genetic Engineering and Human Phenome Institute, School of Life Sciences, Fudan University, Songhu Road 2005, Shanghai, 200438 China

**Keywords:** Coronary artery disease, Atherosclerosis, Endoglin, Genetic interference, Hyperlipidaemia, Drug targets, Endothelial dysfunction

## Abstract

**Background:**

Coronary artery disease (CAD) is a common cardiovascular disease, and abnormal blood lipid metabolism is an important risk factor. Transforming growth factor-ß (TGF-ß) and its receptor (TGF-ßR) can inhibit the release of inflammatory factors through the SMAD pathway-mediated immune response, thereby suppressing the progression of CAD. Endoglin (TGF-ßRIII), a TGF-ßR family homologous receptor protein, is directly involved in the immunoregulatory process, but the exact mechanism is unclear. This study aimed to clarify the pathophysiological effects of endoglin on the development of atherosclerosis and to explore the mechanism of the signalling pathway.

**Methods:**

We downloaded the GEO dataset to perform a functional analysis of SMAD family activity and TGF-ß receptor protein expression in the monocyte expression profiles of patients with familial hyperlipidaemia (FH). The effect of endoglin on endothelial cell proliferation, migration, and apoptosis was examined by disrupting the endoglin gene in human umbilical vein endothelial cells (HUVECs) and validated by western blotting. The related genes and pathways regulated by endoglin were obtained by analysing the sequencing data.

**Results:**

Research has shown that interference with endoglin can promote the proliferation and migration and significantly inhibit the apoptosis of vascular endothelial cells. Interference with endoglin particularly encourages the expression of VEGFB in vascular endothelial cells.

**Conclusion:**

The endoglin gene in vascular endothelial cells regulates the PI3K-Akt, Wnt, TNF, and cellular metabolism pathways by activating the SMAD pathway. RAB26, MR1, CCL2, SLC29A4, IBTK, VEGFB, and GOLGA8B play critical roles. Endoglin interacts closely with 11 proteins such as CCL2 and SEPRINE1, which participate in the vital pathway of plaque formation. Interference with endoglin can alter the course of coronary atherosclerosis.

## Background

Coronary artery disease (CAD) is a common cardiovascular disease, and abnormal lipid metabolism is an important risk factor. Studies have shown that high levels of lipid metabolites produce various cytokines that promote inflammation by injuring the vascular endothelium. Cytokines secrete various inflammatory factors under the action of different signals, mediating the occurrence of acute or chronic inflammatory responses, causing cell adhesion and lipid plaque generation and smooth muscle proliferation and migration, thus initiating the process of atherosclerosis [1, 2]. Transforming growth factor-ß (TGF-ß) and its receptor (TGF-ßR) inhibit the release of inflammatory cytokines through the SMAD2/3/4-mediated immune response, thereby inhibiting the progression of coronary atherosclerosis [3, 4]. TGF-ßRI/II plays an essential role in vector and signal transduction during immunosuppression [5].

Endoglin protein (TGF-ßRIII) is a homologous receptor of the TGF-ßR family. Studies have reported that endoglin levels are significantly reduced in the serum of patients with familial hyperlipidaemia (FH), which can be used as a marker to evaluate the efficacy of drug treatment [6]. Moreover, endoglin on the endothelial cell membrane activates the TGFßRI and TGFßRII-mediated SMAD2 and SMAD3 pathways, respectively, to inhibit the release of downstream inflammatory factors by assisting other receptors [7, 8]. On the other hand, in the hyperlipidaemia mouse model, soluble endoglin was significantly downregulated by statin therapy, but its expression on the aorta was significantly increased [6, 9]. Therefore, it remains unclear among the current academic community whether the protective effect of endoglin is a biological target for atherosclerosis. Given the importance of endoglin in the progression of coronary atherosclerosis [10, 11], its immunological properties in the course of the disease provide an excellent entry point for studying the immunological mechanisms of coronary atherosclerosis.

This study used bioinformatics to analyse the changes in endoglin and TGF-ß in FH patients and combined them with cytological experiments to demonstrate the effect of interfering with endoglin on endothelial cells. The aim of this study was to reveal the physiological role and signalling mechanisms of endoglin in the development of blood lipid-mediated atherosclerosis. This study provides a complementary basis for endoglin as a regulatory target to inhibit abnormal vascular and lipid plaque formation.

## Methods

### GEO database analysis

Gene expression profiling microarray datasets (GSE13985, GSE6088, and GSE6054) of patients with familial hyperlipidaemia (FH) were downloaded from the GEO database (http://www.ncbi.nlm.nih.gov/), with samples derived from 31 normal cell lines, 12 FH cell lines, 13 control participants, and 6 FH patients. In total, 5421 differentially expressed genes (DEGs) were obtained after analysis. The GEO database search keywords were “family hypercholesterolemia,” “*Homo sapiens*,” and “array.”

### Endoglin gene interference experiment

A human umbilical vein endothelial cell (HUVEC) cell line was used for the experiments in this study. HUVECs can be used to examine the underlying molecular events of angiogenesis and atherosclerotic effects [12, 13]. The siRNA interference sequence was designed by querying the endoglin sequence from NCBI and constructed by Shanghai Jima Bios. HUVECs (CinoAsia Co., Ltd. No.CA1256, Shanghai, China) were inoculated into a 24-well plate (1 × 10^5^/well) with 500 μL of DMEM (10% FBS + 1% antibiotic, Thermo Fisher, Shanghai, China) at 37 °C and 5% CO_2_. For transfection, 6 μL of Opti-MEM medium (Thermo Fisher, Shanghai, China) was added to an Eppendorf tube, 4 pmol of endoglin siRNA was added to the medium, and 0.6 μL of HilyMax Transfection Reagent was then added (Dojindo, Japan). The solution was mixed gently and incubated for 15 min at room temperature. The solution was added to the prepared HUVECs, gently shaken and mixed for 6 h in a cell culture incubator. Fluorescence accompanied or parallel well images were used as evidence of transfection efficiency. The interference effect of siRNA on endoglin was confirmed by PCR.

### CCK-8 cell proliferation assay

The cultured HUVECs were inoculated into 96-well culture plates (1*10^4^/well) and cultured overnight in a cell culture incubator for siRNA transfection. Changes in the cell proliferation ability of each group were detected by the CCK-8 method at 0 h, 24 h, and 48 h after transfection. The old solution was discarded, and 95 μL of medium and 5 μL of CCK-8 were added to each well of the 96-well plate to be tested and incubated at 37 °C for 2 h. The OD value of each test well was measured at 450 nm using a microplate reader.

### Transwell assay

Cells in the NC and siRNA groups were inoculated into wells of Matrigel plates containing serum-free medium (24-well plate, 5 × 10^4^ cells/well). The lower chamber was incubated with medium containing 10% foetal bovine serum. After culturing at 37 °C in 5% CO2 for 24 h, the cells in the upper chamber were wiped with a cotton swab, stained with crystal violet, and photographed for counting.

### Annexin-V PI apoptosis detection

The siRNA cells were transfected. After incubation for 6 h, the old medium in the culture plate was removed, fresh complete medium was added, and the cells were cultured in a cell culture incubator for 48 h. Cells were collected by trypsin digestion without EDTA and washed once each with PBS and binding buffer. Cells were resuspended in binding buffer, and the cell concentration was adjusted to 1*10^6^/mL. Then, 100 μL of cell suspension was taken, and 5 μL of Annexin V-FITC and 5 μl of propidium iodide were added and mixed. The reaction was carried out for 15–20 min at room temperature and protected from light. Another 500 μL of binding buffer was then added and mixed. Finally, cells were analysed by fluorescence-activated cell sorting with FITC and PI channels.

### RNA extraction and RT–qPCR

Total RNA was extracted from cells using TRIzol reagent, and the purity of extracted RNA was confirmed using a NanoDrop 1000. The primers were designed using Primer Premier 5.0 software (Premier Biosoft International, Palo Alto, CA, USA) and synthesized by Generay Biotech Co., Ltd. RT–qPCR was performed using the KAPA SYBR Green Supermix PCR kit with the AriaMx Real-Time PCR System (both from Agilent Technologies, Inc. Santa Clara, CA, USA). The 2^-ΔΔCT^ method was used for semiquantitative analysis [14]. Table 1 shows the primer and siRNA information.

**Table 1:** Primer and siRNA information

Primer	Sequence (5′-3′)
endoglin-siRNA sense	GGCCAGCAUUGUCUCACUUTT
endoglin-siRNA antisense	AAGUGAGACAAUGCUGGCCTT
Homo-endoglin-F	GATCCAGACAAAGTGTGCCG
Homo-endoglin-R	AGGATATTGACCACCGCCTC
Homo-SMAD2-F	CCAGGTCTCTTGATGGTCGT
Homo-SMAD2-R	TTAGGATCTCGGTGTGTCGG
Homo-SMAD3-F	ATAACTTGGACCTGCAGCCA
Homo-SMAD3-R	ACATTGGAGAGCAGCCCTAG
Homo-SMAD4-F	GCTGCTGGAATTGGTGTTGA
Homo-SMAD4-R	CTTCGTCTAGGAGCTGGAGG
Homo-BCL2-F	TTCTTTGAGTTCGGTGGGGT
Homo-BCL2-R	CTTCAGAGACAGCCAGGAGA
Homo-SERPINE1-F	ACTGGAAAGGCAACATGACC
Homo-SERPINE1-R	TGACAGCTGTGGATGAGGAG
Homo-CCL2-F	TCTGTGCCTGCTGCTCATAG
Homo-CCL2-R	CAGATCTCCTTGGCCACAAT

### Western blotting

The total protein of cells was extracted at 0 h, 24 h, 48 h, and 72 h, and the protein concentration was determined by the BCA method. The protein samples were separated by electrophoresis on SDS-PAGE gels and transferred to nitrocellulose membranes. After blocking, the cells were incubated with SMAD3 (Abcam, ab40854), BCL2 (Abcam, ab182858), and GAPDH (PTG, 60004–1-lg) antibodies overnight at 4 °C. After incubation with the secondary antibody for 1 h at 20 °C, the membranes were washed three times. An ECL detection system measured the immunoreactivity of the bands, which were quantified using ImageJ software (NIH, Bethesda, MD, USA).

### RNA database and sequencing

The cell culture and siRNA interference methods were the same as above, and the cells were collected at 0 h, 24 h, 48 h, and 72 h for RNA sequencing. Each group was repeated three times. Cellular RNA was extracted and reverse transcribed for PCR amplification. The PCR amplification product was purified and quality-controlled in a 96-well plate. The above samples were subjected to molar conversion, and each sample was diluted to a concentration of 2 nM and mixed 1:1 for each sample. After mixing, 10 μL was sent for sequencing. A HiSeq 2500 sequencer was used, and the sequencing mode was 2 × 150 bp read length and 250 M read number.

### RNA sequencing data analysis and statistical analysis

Raw data were processed using Trimmomatic to remove sequencing linker sequences and low-quality read lengths. In this analysis, gene counts greater than 1 were defined as expressed genes. The read count was then normalized to the TPM value for log2 conversion for the “newSCESet” function conversion of the “scater” package in R. Differentially expressed genes (DEGs) were identified by calculating the fold changes and *p* values between the two groups. Genes with changes of more than two fold with *P* < 0.05 were set as the standard for DEG selection, and the “stat” tool in R was used. A DEG intersection was generated with the “Venn Diagram” online tool, and ‘pheatmap’ (v.1.0.12) was used to draw a heatmap. Path analysis was performed with “KEGG profile.” Gene Ontology (GO) analysis was performed by DAVID (v6.7). Protein-Protein Interaction (PPI) network was performed by Cytoscape (v3.8.2). Histograms were made using GraphPad software, and Student’s t-test were conducted to measure the significance of differences between groups.

## Results

### Bioinformatics analysis from FH mononuclear cells

**Fig. 1:**
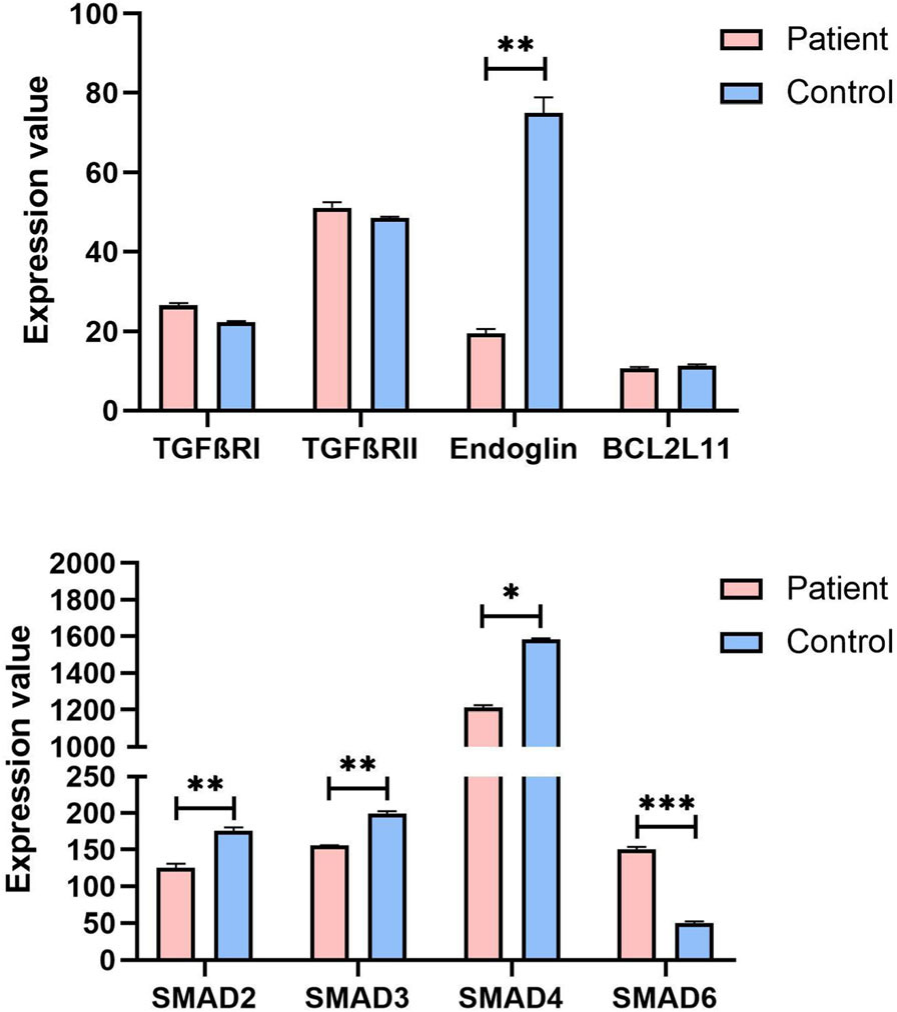
Exploring gene expression changes in FH patients using the GEO dataset. Endoglin expression was significantly down-regulated in FH patients, and TGFβR1/2 itself did not change significantly. In the downstream SMAD family, SMAD2, SMAD3, and SMAD4 were down-regulated in patients, while SMAD6 was up-regulated significantly (*P < 0.05, **P < 0.01, ***P < 0.001)

Analysis of monocyte expression profile datasets from 6 FH patients and 13 control participants revealed a negative correlation between endoglin expression and disease in FH patients. Meanwhile, the TGFßRI/II expression level did not change significantly, while downstream SMAD2, SMAD3, SMAD4, and BCL2L11 were downregulated considerably, and SMAD6 was significantly upregulated in the patients (Fig. 1).

### Endoglin gene interference and subsequent experiments in normal endothelial cells

**Fig. 2:**
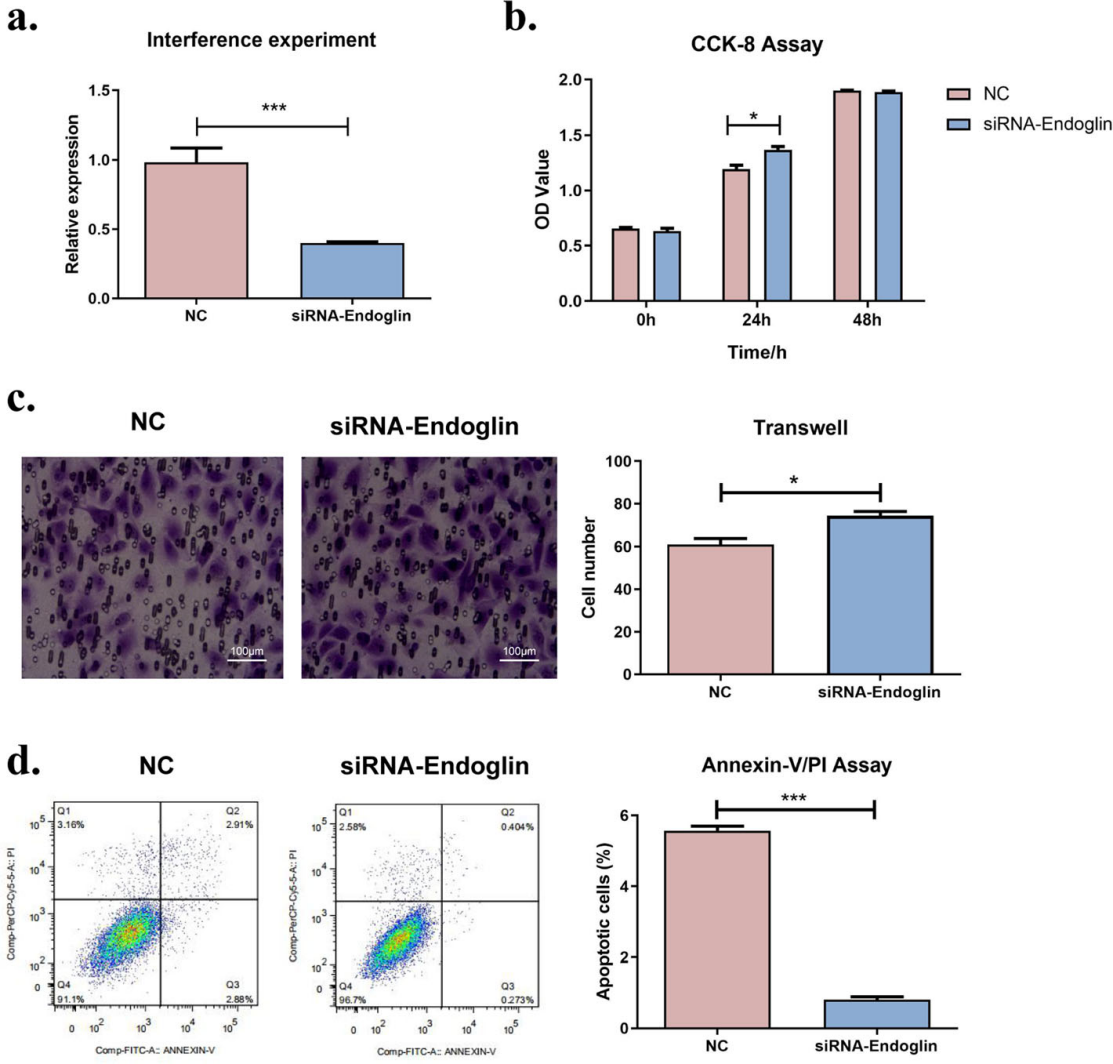
Endoglin impedes endothelial cell growth progression. **a** Endoglin gene interference experiments shown that siRNA can be used in subsequent experiments; **b** CCK8 assay demonstrated that interference of endoglin gene could promote HUVEC proliferation(* *P* < 0.05, *** *P* < 0.001); **c** Transwell cell migration assay demonstrates that migration of HUVEC can be promoted by interference with the endoglin gene; **d** Apoptosis experiments in av./pi cells demonstrated that the inhibition of endoglin gene significantly inhibited apoptosis of HUVEC (*P < 0.05, **P < 0.01, ***P < 0.001)

As shown in Fig. 2a, the results of real-time PCR showed that the expression of endoglin in the siRNA group was significantly lower than that in the NC group (*P* < 0.05), which could be used for subsequent validation experiments.

Compared with the NC group, the proliferation ability of the siRNA group was significantly enhanced after 24 h, indicating that endoglin could promote the proliferation of endothelial cells after interference (*P* < 0.05, Fig. 2b). There was no significant difference in the cell proliferation levels between the two groups at 48 h, likely because the cells in all culture wells were confluent, leaving no room for proliferation.

Transwell assays indicated that the migration ability of the siRNA group was significantly increased after transfection (*P* < 0.05). The results showed that interference with the endoglin gene could promote the migration of HUVECs (Fig. 2c). Apoptosis in the siRNA group was significantly decreased after transfection compared with that in the NC group (*P* < 0.001). This result indicates that interference with the endoglin gene significantly inhibits the apoptosis of HUVECs (Fig. 2d).

**Fig. 3:**
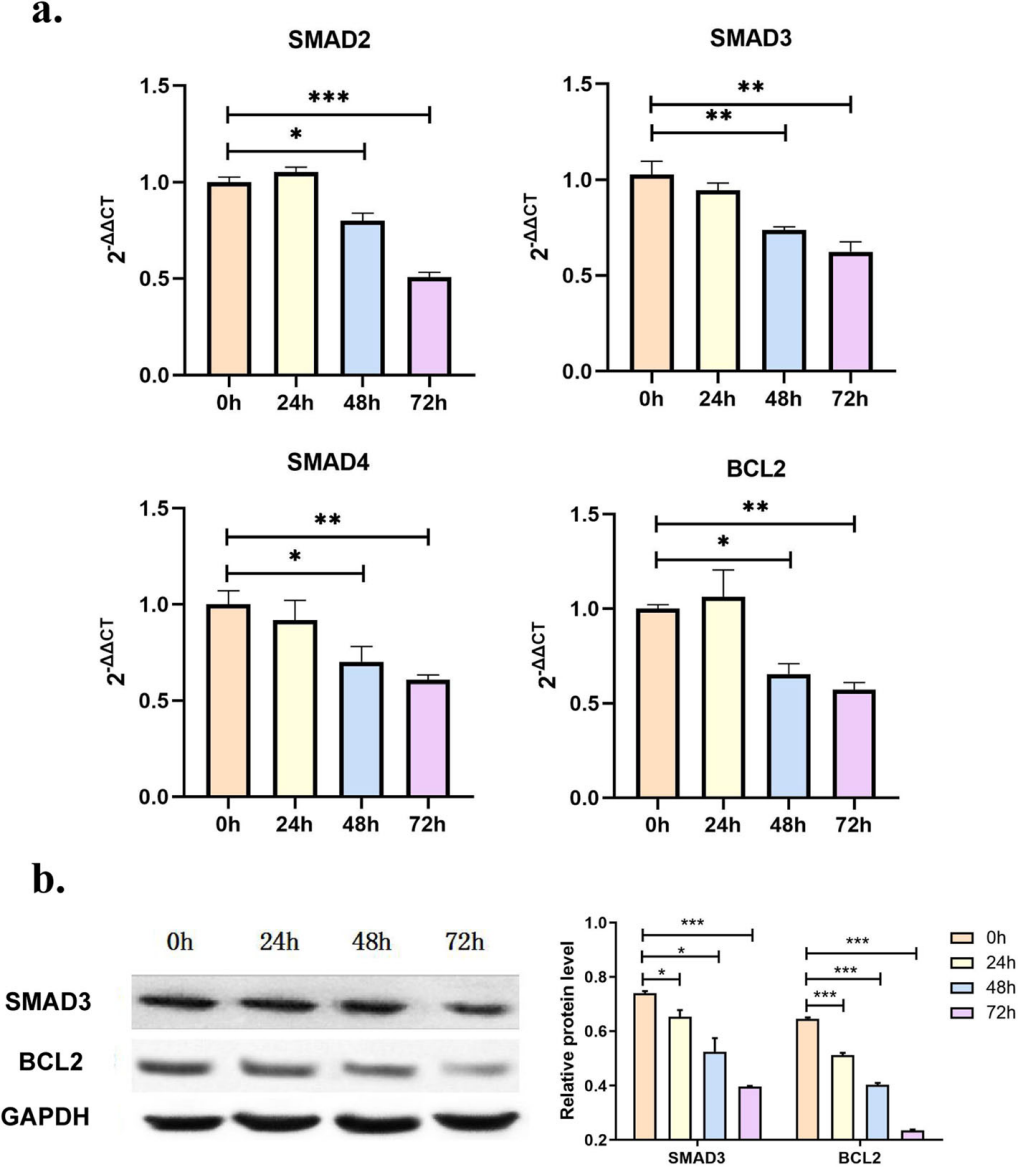
Changes in gene levels in cells after endoglin silencing. **a** Cellular fluorescence quantitative PCR detection. Compared with the 0 h group, the gene expressions of SMAD2, SMAD3, SMAD4, and BCL2 were significantly decreased in the 48 h and 72 h groups; **b** Western blotting analysis showed that the expression of Smad3 and BCL2 protein in the 72 h group was significantly lower than that in the 0 h group after endoglin gene silencing (*P < 0.05, **P < 0.01, ***P < 0.001)

The expression levels of SMAD2, SMAD3, SMAD4, and BCL2 in endothelial cells after endoglin gene interference were detected by real-time PCR. The expression of SMAD2, SMAD3, SMAD4, and BCL2 was significantly decreased in the 48 h and 72 h groups (Fig. 3a). Western blotting analysis of the proteins at various time points showed that Smad3 and BCL2 protein expression in the 72 h group was significantly lower than that in the 0 h group after endoglin silencing (Fig. 3b).

### RNA sequencing analysis

**Fig. 4:**
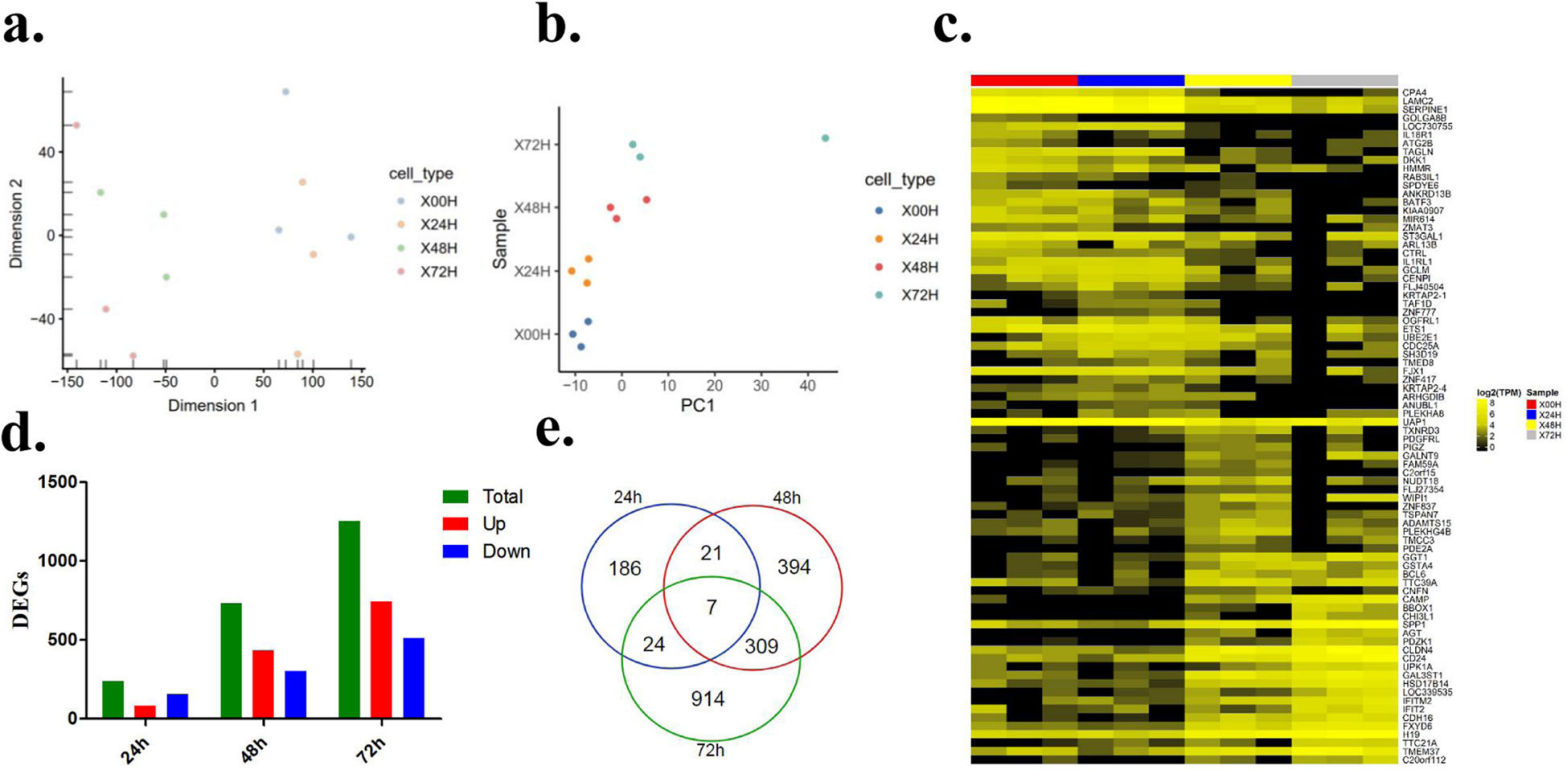
Differentially expressed genes regulated by endoglin. **a** T-SNE cluster analysis shows that the differences between the groups are significant, and the samples in the group are reproducible; **b** At the PC1 level, the 0 h group (X00H) and the 72 groups (X72H) group have the largest difference, and the sample also changes with time; **c** HCA heat map analysis shows that the sample has good repetitiveness; **d** Number of differential genes (DEGs) compared with NC and OC groups; **e** Venn plots show the number of identical differentially expressed genes obtained in different groups compared to the 0 h group

T-SNE cluster analysis showed that the differences between the groups were significant (Fig. 4a). At the PC1 level, the 0 h group (X00H) and 72 h group (X72H) had the largest difference, and the samples also changed with time (Fig. 4b). In hierarchical cluster analysis (HCA), except for the 0 h and 24 h groups, the samples from different experimental groups were well separated and had good reproducibility (Fig. 4c). The results of the DEG analysis showed that the number of allogeneic genes in the 24 h, 48 h, and 72 h groups was 238, 731, and 1254, respectively, compared with the 0 h group. The number of DEGs also indicated that the differences increased over time (Fig. 4d). Venn intersection gene analysis showed seven DEGs between the groups compared with the 0 h group, namely, RAB26, MR1, CCL2, SLC29A4, IBTK, VEGFB, and GOLGA8B (Fig. 4e).

**Fig. 5:**
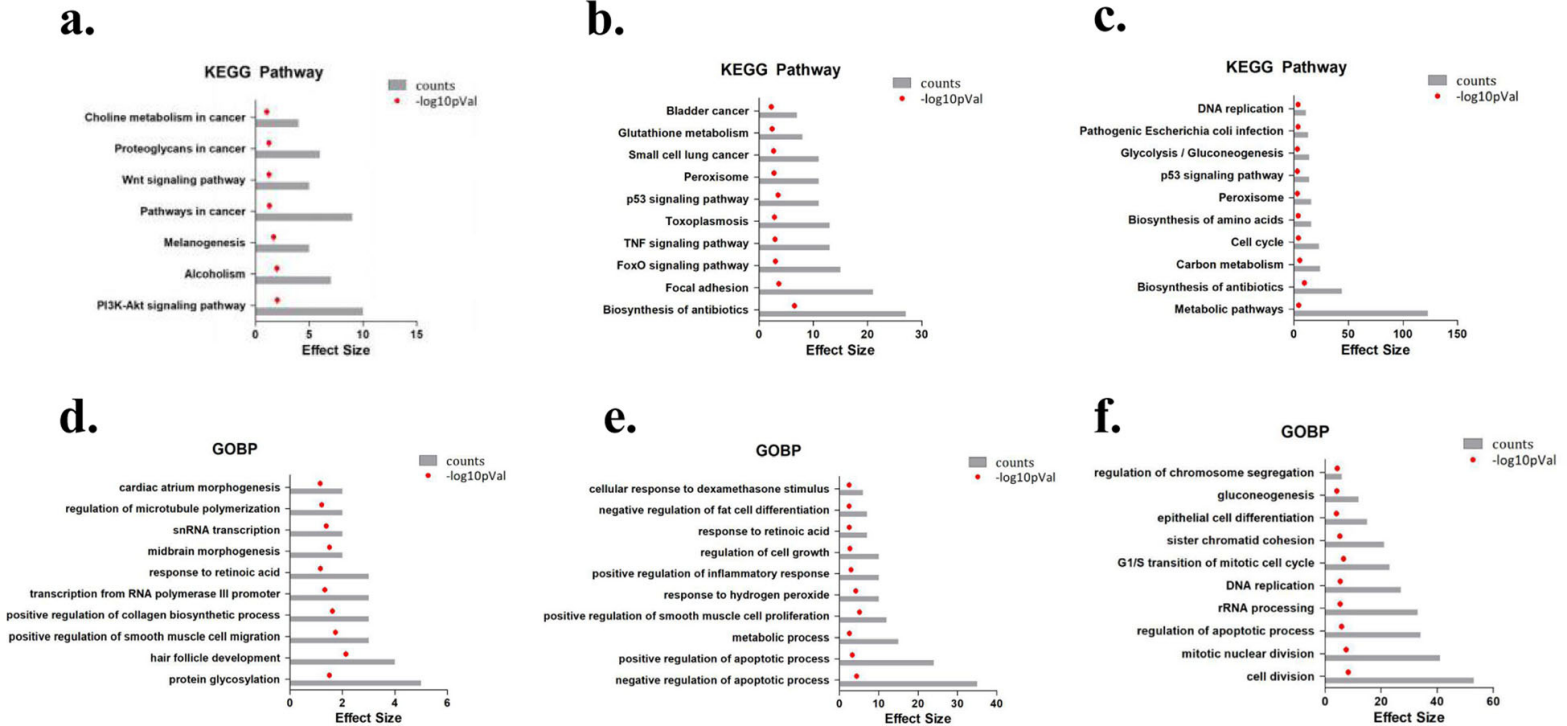
Related signalling pathways regulated by endoglin. **a** Analysis of the trend gene KEGG pathway between 24 h and 0 h; **b** Analysis of the trend gene KEGG pathway between 48 h and 0 h; **c** analysis of trend gene KEGG pathway between 72 h and 0 h; **d** GO function analysis of trend genes between 24 h and 0 h; **e** GO function analysis of trend genes between 48 h and 0 h; **f** GO function analysis of trend genes between 72 h and 0 h

Further analysis of the functions and pathways that the DEGs are involved in at different periods revealed that the pathways included the PI3K-Akt and Wnt pathways (Fig. 5a, 24 h), TNF and FoxO pathways (Fig. 5b, 48 h), and cellular metabolism pathways (Fig. 5c, 72 h). GO functional analysis showed that the DEGs were mainly involved in related functions, such as cell metabolism, apoptosis, cell division and cell cycle progression (Fig. 5d, e, f).

**Fig. 6:**
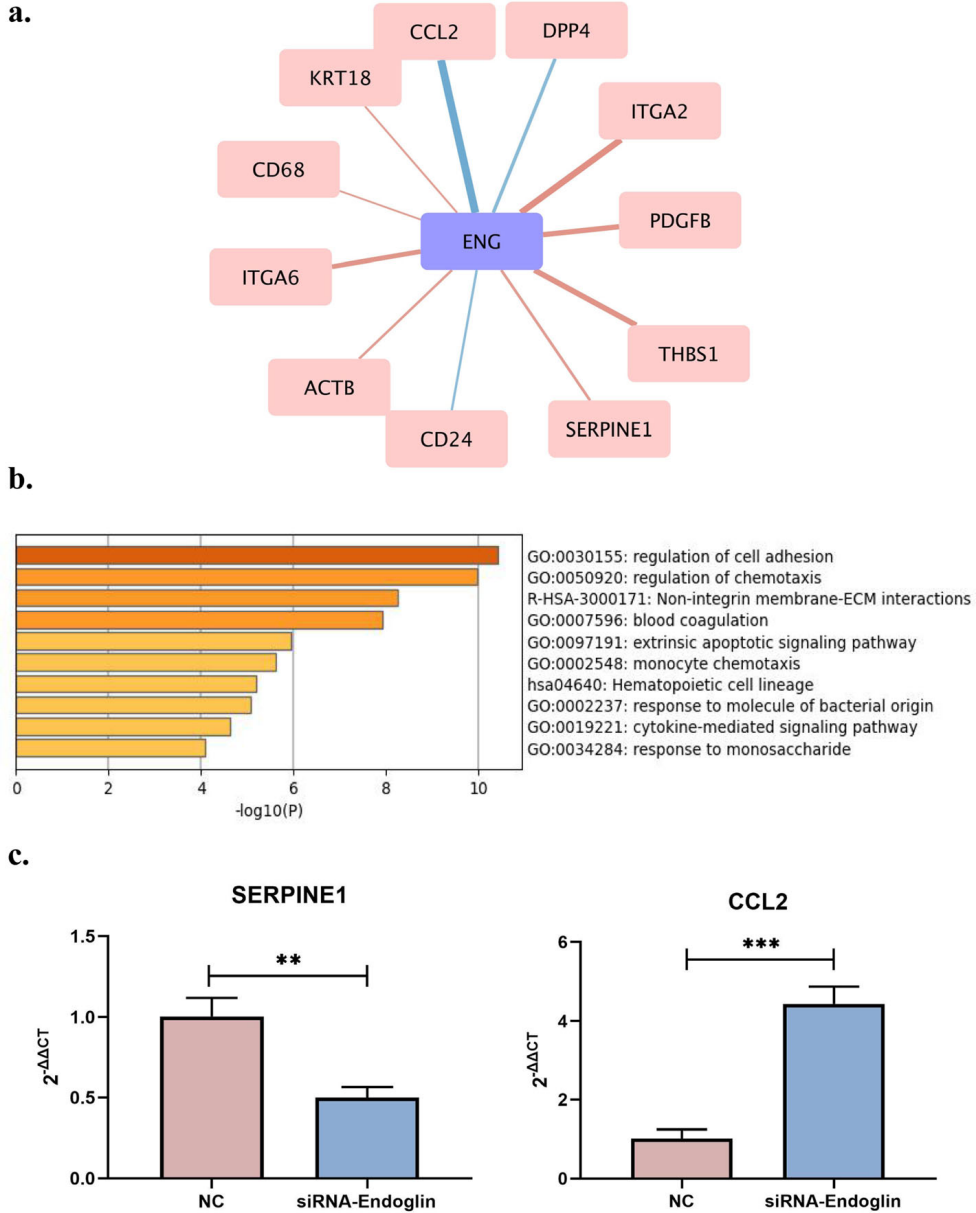
Endoglin interacts closely with 11 proteins and participates in the vital pathway of plaque formation. **a** Protein-Protein Interaction (PPI) network elucidated 11 proteins interacts closely with endoglin; **b** GO function analysis of PPI network proteins; **c** Gene expressions of CCL2 and SERPINE1

Next, all DEGs were used to construct PPI network with endoglin by the ‘string’ database, and the interacting proteins were filtered at *P* < 0.05. PPI network was shown in Fig. 6a, with red lines indicating positive correlations and blue indicating negative correlations. These proteins are involved in cell adhesion, ECM, and blood coagulation, which are important mechanisms for plaque formation (Fig. 6b). The results of PCR showed that the pro-inflammatory factor CCL2 was up-regulated, while the anti-migration gene SERPINE1 was down-regulated after endoglin silencing (48 h, Fig. 6c).

## Discussion

Endoglin is expressed in various tissues, such as mouse and human atherosclerotic vascular endothelial cells and smooth muscle cells [15]. It is related to the expression of eNOS in endothelial cells (which can repair the blood vessel wall), plaque neovascularization, collagen production, and atherosclerotic plaque damage [16]. As a TGF-ßR family homologous receptor, endoglin protein (TGF-ßRIII) is directly involved in the activation and signalling of the SMAD2 and SMAD3 pathways by TGF-ßRI/II [8, 9, 17]. However, the specific mechanism is unclear, and the interaction mechanism between endoglin protein and other signalling pathways has rarely been reported. In this study, endoglin, SMAD2, SMAD3 and SMAD4 were downregulated in familial hyperlipidaemia (FH) patients, but the expression levels of TGFßRI and TGFßRII did not change significantly. Changes in high-fat metabolites suggest the importance of endoglin for immune inflammatory responses mediated by the TGF-ßRI/II-SMAD pathway.

In this study, gene expression profiling, SMAD family activity functional analysis, and TGF-ß receptor protein expression were performed in monocyte expression profile datasets of FH patients. Interference with the endoglin gene in HUVECs; cell proliferation, migration, and apoptosis experiments; PCR and western blotting verification; and RNA library sequencing were performed. Analysis of RNA sequencing data revealed related genes and pathways regulated by endoglin.

TGF-ßRII phosphorylates TGF-ßRI through a conformational change following autophosphorylation of serine/threonine in the GS structural domain of the cytoplasmic region, which in turn initiates kinase activity and recruitment of SMAD family proteins. With the help of the SMAD receptor activation anchor (SARA), SMAD2 and SMAD3 are phosphorylated and then form a complex with SMAD4, which translocates to the nucleus. Together with other transcription factors, the promoters of specific genes are regulated to inhibit the release of inflammatory factors. In this process, SMAD6 interferes with the phosphorylation of SMAD2 protein, which inhibits the heterodimerization of SMAD4 protein and the formation of the SMAD4 complex, interfering with the initiation of inflammatory factor release [4, 5]. BCL2L11, a member of the BCL2 family, is involved in the initiation of programmed apoptosis. Downregulation of the expression of this molecule implies that immune cells enter an activation and proliferation state. This study showed that the endoglin gene, after interference, can promote the proliferation and migration of vascular endothelial cells and significantly inhibit the apoptosis of vascular endothelial cells. It also led to the downregulation of SMAD2, SMAD3, and SMAD4 expression, resulting in loss of function of the SMAD2/3/4 complex, which could not negatively regulate immune-activated transcription factors. This further led to a significant downregulation of BCL2L11 expression, resulting in cells no longer being regulated by apoptosis and proliferating significantly. Significantly promoting VEGFB expression in vascular endothelial cells can induce angiogenesis and increase permeability and can also promote the migration of mononuclear macrophages into AS plaques, secretion of TNF-a, and further promotion of plaque internal angiogenesis.

Similar to these results, previous studies have shown that endoglin regulates PI3-kinase/Akt trafficking and signalling to alter endothelial capillary stability during angiogenesis [18]. Endoglin integrates BMP and Wnt signalling to induce haematopoiesis through JDP2 [19]. Endothelial cell inflammation and barriers are regulated by the Rab26-mediated balance between β2-AR and TLR4 in pulmonary microvessel endothelial cells [20].

PPI network results elucidated 11 proteins interacts closely with endoglin, namely CCL2, DPP4, ITGA2, PDGFB, THBS1, SERPINE1, CD24, ACTB, ITGA6, CD68, and KRT18. CCL2 is thought to play a key role in recruiting monocytes to the vascular endothelium where the adherence of monocytes is one of the earliest events in atherogenesis [21]. SERPINE1 controlled degradation of blood clots [22], inhibit plasmin generation and thereby stabilize plaque [23].

Coronary atherosclerosis is a metabolic disorder characterized by hyperlipidaemia and chronic inflammation. In this study, bioinformatics analysis revealed changes in endoglin and TGF-ß in the course of blood lipid-mediated coronary atherosclerosis. These experimental results complement the potential target genes and molecular pathways that link endoglin to CAD disease progression.

### Study strengths and limitations

The strengths of this study are the discovery and demonstration of the protective role of endoglin in hyperlipidaemia and abnormal plaque formation through activation of the SMAD pathway. The results highlight that endoglin can impede endothelial dysfunction and atherosclerosis and explored the relevant molecular pathways. There are also some limitations of this study that need to be addressed. First, the endoglin agonist group was not designed as a positive control. Second, an animal model of high-fat diet intervention was not constructed for in vivo validation.

## Conclusion

In summary, the above data support the notion that the endoglin gene in vascular endothelial cells regulates the PI3K-Akt, Wnt, TNF, and cellular metabolism pathways through activation of the SMAD pathway. RAB26, MR1, CCL2, SLC29A4, IBTK, VEGFB, and GOLGA8B may play critical roles in atherosclerotic plaque formation. Endoglin interacts closely with 11 proteins such as CCL2 and SEPRINE1, which participate in the vital pathway of plaque formation. Designing targeted drugs for endoglin and downstream genes to treat hyperlipidaemia may improve endothelial dysfunction and reduce the incidence of plaque formation and complications.

## Data Availability

The datasets used and/or analysed during the current study are available from the corresponding author on reasonable request.
